# Alterations of Dopamine-Related Transcripts in A11 Diencephalospinal Pathways after Spinal Cord Injury

**DOI:** 10.1155/2021/8838932

**Published:** 2021-01-15

**Authors:** Shunyi Zhao, Jaclyn H. DeFinis, Shaoping Hou

**Affiliations:** ^1^Marion Murray Spinal Cord Research Center, Department of Neurobiology & Anatomy, Drexel University College of Medicine, Philadelphia, PA, USA; ^2^Department of Pharmacology & Physiology, Drexel University College of Medicine, Philadelphia, PA, USA

## Abstract

The diencephalic A11 nuclei are the primary source of spinal dopamine (DA). Neurons in this region project to all levels of the spinal cord. Traumatic spinal cord injury (SCI) often interrupts descending and ascending neuronal pathways and further elicits injury-induced neuronal plasticity. However, it is unknown how A11 neurons and projections respond to SCI-induced axotomy. Based on preliminary observation, we hypothesized that A11 DA-ergic neurons rostral to the lesion site might change their capacity to synthesize DA after SCI. Adult rats received a complete spinal cord transection at the 10th thoracic (T10) level. After 3 or 8 weeks, rostral (T5) and caudal (L1) spinal cord tissue was collected to measure mRNA levels of DA-related genes. Meanwhile, A11 neurons in the brain were explicitly isolated by laser capture microdissection, and single-cell qPCR was employed to evaluate mRNA levels in the soma. Histological analysis was conducted to assess the number of A11 DA-ergic neurons. The results showed that, compared to naïve rats, mRNA levels of tyrosine hydroxylase (TH), dopamine decarboxylase (DDC), and D_2_ receptors in the T5 spinal segment had a transient decrease and subsequent recovery. However, dopamine-*β*-hydroxylase (DBH), D_1_ receptors, and DA-associated transcription factors did not change following SCI. Furthermore, axon degeneration below the lesion substantially reduced mRNA levels of TH and D_2_ in the L1 spinal segment. However, DDC transcript underwent only a temporary decrease. Similar mRNA levels of DA-related enzymes were detected in the A11 neuronal soma between naïve and SCI rats. In addition, immunostaining revealed that the number of A11 DA neurons did not change after SCI, indicating a sustention of capacity to synthesize DA in the neuroplasm. Thus, impaired A11 diencephalospinal pathways following SCI may transiently reduce DA production in the spinal cord rostral to the lesion but not in the brain.

## 1. Introduction

The synthesis of dopamine (DA) is initiated by the rate-limiting enzyme, tyrosine hydroxylase (TH), which converts tyrosine to the DA precursor L-DOPA. Subsequently, dopamine decarboxylase (DDC) removes the carboxyl group from L-DOPA to produce DA. In noradrenergic cells, DA is further catalyzed to norepinephrine (NE) by dopamine-*β*-hydroxylase (DBH) [1]. In DA-ergic neurons, the synthesis of DA occurs in both the cell soma and axoplasm, and so, TH is stored in neuronal bodies and presynaptic axonal terminals [2]. Previous studies showed that axonal TH protein can be transported from the neuroplasm or synthesized by axonal translation mechanisms. The presence of axonal TH mRNA was originally suggested by the detection of TH transcript in the neocortex, striatum, and cerebellum where there are catecholaminergic axons but no cell bodies [3, 4]. Recently, researchers isolated pure axonal TH mRNA from cultured sympathetic neurons, suggesting that TH mRNA can be transported to neuronal axons for local DA synthesis. Furthermore, transfection of exogenous TH mRNA into these axons increased DA synthesis [5]. This indicates that axonal projections contain the applicable milieu for transcription of TH.

DA neurons are mainly distributed in the substantia nigra (SN), ventral tegmental area (VTA), and hypothalamus of the brain. They project to a variety of areas but most of their extensions are restricted within the brainstem and cerebral cortex [6]. In particular, diencephalic A11 DA neurons project to the spinal cord as the primary source of spinal DA [7, 8]. Functionally, these projections to the cord have been shown to modulate both pain and locomotor networks. It was demonstrated that electrical stimulation of the A11 nuclei suppresses nociceptive responses of neurons in the dorsal horn through D_2_-like receptors [9]. Optogenetic activation of channelrhodopsin-2 (ChR2) transfected A11 neurons can initiate locomotion and enhance motor activity [10]. Accordingly, A11 neurons have been used as a therapeutic target for treating restless leg syndrome (RLS), which is characterized by abnormal limb sensations with an uncontrolled desire to move legs [11–13].

Traumatic spinal cord injury (SCI) interrupts connections between higher regulatory centers and spinal afferent/efferent components. It causes neurodegeneration, impaired protein synthesis, and morphological alterations in axons. In the spinal cord, supraspinal projections have alterations at different levels in response to an injury. Below the lesion, axons undergo Wallerian degeneration that begins with axonal swelling followed by membrane beading and fragmentation to cleave decentralized axons [14, 15]. In the rostral region, denervated fibers may sprout in order to make new connections to establish an intraspinal circuit [16]. Although neural degeneration caudal to the lesion has been extensively studied, the rostral portion is less understood. At present, it is still debated whether neurons die due to axotomy. For example, one study reported that about 20% of cortical pyramidal neurons undergo apoptosis following injury to the corticospinal tract (CST) [17], while succeeding research failed to repeat the results when using the same methods [18, 19]. Nevertheless, axotomized CST cells exhibit impaired protein synthesis machinery in the soma, which includes destruction of endoplasmic reticulum and degeneration of mRNA [20].

It is unknown how the diencephalospinal A11 DA-ergic system responds to the injury. Based on our primary observations, we hypothesized that A11 neurons may change the capacity to synthesize DA in the spinal cord rostral to the lesion site. To understand the plasticity of supraspinal DA-ergic pathways, in the present study, we explored whether DA-related gene expression is altered at different levels of the spinal cord and brain following SCI.

## 2. Materials and Methods

### 2.1. Animals and Genotyping

A total of 33 adult female Wistar rats (2-3 months old, weighing 200–250 g) and 14 adult female hTH-eGFP transgenic rats (Taconic, NTac:SD-Tg (TH-EFGP) 24Xen) were used in these experiments. Only female rats were included in consideration of bladder care procedures after SCI, which are much more extensive for males. The guidelines of the Institutional Animal Care and Use Committee at Drexel University and National Institutes of Health on animal care and housing were strictly followed to minimize both the number of animals used and any potential suffering.

Adult hTH-eGFP transgenic rats were bred in-house. GFP^+^ pups were identified by PCR and subsequent electrophoresis to test the existence of a 264 bp eGFP open reading frame (eGFP ORF, Table 1) from tail tip lysates. Collected tail tissues were dissolved in 150 *μ*L of lysis buffer (50 mM Tris (pH 8.0), 10 mM EDTA, 100 mM NaCl, 0.5% SDS, and 0.5 mg/mL proteinase K (Sigma-Aldrich)) at 55°C overnight to extract DNA. When tissue was fully digested the following day, 0.5 *μ*L of lysis solution was used as template for PCR. Following this, agarose gel electrophoresis (NuSieve 3 : 1 agarose) with ethidium bromide (Promega) staining was applied to visualize the presence of eGFP. Immediately after electrophoresis, the gel was examined under ultraviolet transillumination and imaged using GeneSnap software (Syngene).

### 2.2. Spinal Cord Surgery

Rats underwent a complete spinal cord transection at the 10^th^ thoracic (T10) level to disrupt descending and ascending pathways. A total of 28 rats received SCI, and 16 nonoperated naïve rats served as a control. Rats were anesthetized with 4% isoflurane in oxygen, and 2% isoflurane was used during surgery to keep animals anesthetized on heating pads. A 2 cm midline incision was made on the back of the skin at the midthoracic level, and a T8 laminectomy was performed to expose the dorsal part of the T10 spinal segment. The spinal cord was completely transected using a no. 11 blade and confirmed visually at the time of surgery. After surgery, animals were subcutaneously administered cefazolin (10 mg/kg, West-ward), 3 mL Lactated Ringer's solution (VEDCO), and buprenorphine hydrochloride (0.1 mg/kg, Par). Rats survived for an additional 3 (subacute) or 8 weeks (chronic). Postsurgical animal bladder care was manually conducted at least three times a day until sacrifice.

### 2.3. Tissue Processing and Single-Cell Laser Capture Microdissection (LMD)

Three or 8 weeks after SCI, fresh tissue samples were collected for real-time qPCR. Rats were anesthetized by intraperitoneal (*i.p*.) injection of 0.2 mL Euthasol (VEDCO) and sacrificed by decapitation. The brain along with 2 spinal segments which included T5 (above injury) and L1 (below injury) levels was immediately dissected [21]. The brain was stored at -80°C until sectioning and LMD was performed. Spinal segments were also stored at -80°C for subsequent RNA extraction and real-time qPCR.

**Figure 1:**
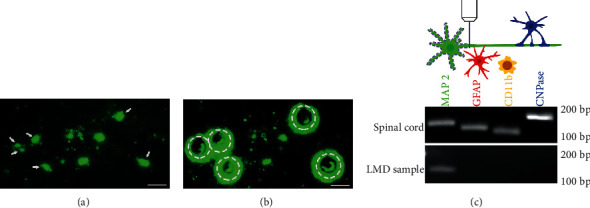
Single-cell laser capture microdissection (LMD) of A11 DA-ergic neurons. (a, b) Isolation of A11 neurons using LMD in fresh brain sections of hTH-eGFP transgenic rats. GFP-expressing A11 DA-ergic neurons (arrows) can be directly detected under a fluorescent microscope (a) and captured by laser. Following successful isolation, empty circles are present where DA-ergic cells were captured from A11 nuclei (b). (c) Representative data shows high purity of neurons in LMD samples. The electrophoresis gel shows the presence of MAP2 (green, neuronal marker), GFAP (red, astrocytic marker), CD11b (yellow, microglia marker), and CNPase (blue, oligodendrocytic marker) in whole spinal cord tissue lysate. However, only MAP2 was present for LMD samples, indicating that a high purity of neurons was obtained when utilizing LMD to isolate A11 DA-ergic cells. Scale bars: 50 *μ*m.

The A11 nuclei contain hundreds of DA-ergic neurons, and the region itself borders other DA producing regions, including the A9 (substantia nigra, SN), A10 (ventral tegmental area, VTA), and A13 in the zona incerta [22]. To specifically isolate A11 neurons, brains used for LMD were freshly collected from hTH-eGFP rats, and coronal sections with a thickness of 16 *μ*m were obtained using a cryostat (Microm) at -20°C. Once A11 nuclei were identified, ten sections were continuously mounted on RNase-free polyethylene naphthalene (PEN) membrane slides (Invitrogen), and GFP^+^ DA neurons were individually dissected using a fluorescent LMD system (Leica) under a 40x objective (Figures 1(a) and 1(b)). The entire procedure was completed within 1 h to preserve the quality of RNA. At least 50 cells were captured per animal and immersed into 100 *μ*L of lysis solution that was provided by a RNAqueous-Micro total RNA isolation kit (Invitrogen). Samples were stored at -80°C.

### 2.4. RNA Extraction, mRNA Amplification, and cDNA Synthesis

For LMD cell samples, RNA was extracted after mixing 1.25 volumes of 100% RNase-free ethanol to the lysate. About 15 *μ*L of eluted mRNA was collected in a 1.5 mL tube. Because each cell contained only ~2-100 pg of RNA [23], the RNA concentration of 50 neurons was too low for reverse transcription. Therefore, RNA amplification was performed for cDNA synthesis using a MessageBooster kit (Lucigen) that is specifically designed to work with very low levels of RNA (<500 pg). Finally, cDNA was stored at -20°C for further real-time qPCR.

For spinal tissue samples, RNA was isolated by E.Z.N.A. RNA total kit (Omega). Samples weighing about 30 mg were homogenized, and mRNA was extracted, eluted, and collected. Lastly, 3 *μ*L of RNA was aliquoted to evaluate RNA concentration and purity using a NanoDrop (Thermo Fisher). RNA concentration and purity were routinely measured at 230, 260, and 280 nm wavelength to determine the OD260/280 and OD260/230 ratio. Purified RNA from tissue samples was converted to cDNA by reverse transcription using an iScript cDNA synthesis kit (Bio-Rad). Diluted cDNA was then stored at -20°C or used for real-time qPCR.

### 2.5. PCR and Real-Time qPCR Analysis

Tissue samples from the brain contain neuronal and nonneuronal components, such as astrocytes, microglia, and oligodendrocytes. To ensure the neuronal specificity of the LMD procedure, we examined several cell type markers by PCR and electrophoresis, including microtubule-associated protein 2 (MAP2) for neurons, glial fibrillary acidic protein (GFAP) for astrocytes, cluster of differentiation molecule 11b (CD11b) for microglia, and 2′,3′-cyclic-nucleotide 3′-phosphodiesterase (CNPase) for oligodendrocytes (Table 1). The PCR products were visualized by agarose gel electrophoresis. As a result, all cell type markers were present in spinal tissue samples while LMD samples included only the neuronal marker MAP2, indicating that the majority of samples isolated are largely A11 cells (Figure 1(c)).

A SYBR Green Supermix Kit (Bio-Rad) was used for real-time qPCR. The analysis for spinal tissue samples was performed with the following primers: (1) 18s ribosomal RNA, (2) TH, (3) DDC, (4) DBH, (5) LIM homeobox transcription factor 1 beta (Lmx1b), (6) pituitary homeobox 3 (Pitx3), (7) D_1_, and (8) D_2_ genes (Table 1). For neurons isolated from the A9, A10, and A11 nuclei, selected target genes included (1) 18s ribosomal RNA, (2) TH, (3) DDC, (4) nuclear receptor related-1 protein (Nurr1), (5) D_2_, and (6) VMAT2. The threshold cycle (Ct) value was examined using the CFX96 real-time qPCR detection system (Bio-Rad). Additionally, all Ct values that were above 40 cycles were excluded during the data analysis. For data normalization, the Ct value of 18s ribosomal RNA was determined as the reference for all samples, and the relative quantification method (2^−ΔΔCt^) was used. Duplication was used to reduce the variability within samples.

### 2.6. Immunohistochemistry and Quantification

Animals were sacrificed for histological analysis 3- or 8-week post SCI. After euthanization with Euthasol, a transcardial perfusion was performed with 200 mL of 0.1 M phosphate-buffered saline (PBS, pH 7.4) and then 200 mL of 4% paraformaldehyde (PFA) in PBS. The brain was dissected, postfixed in 4% PFA overnight, and cryoprotected in 30% sucrose over 2 nights at 4°C. Before cryostat sectioning, tissue samples were embedded in optimum cutting temperature (OCT) compound. Coronal sections of the brain (35 *μ*m) were obtained as six series using a cryostat (Microm).

One series of free-floating brain sections were washed 3 times in tris-buffered saline (TBS) and immersed in blocking buffer (TBS containing 5% donkey serum and 0.5% Triton X-100) for 30 min. Subsequently, sections were incubated in solution containing primary antibodies of TH (rabbit, 1 : 1000, Millipore, AB152) and GFP (chicken, 1 : 1000, Aves, GFP-1020) overnight at 4°C. Following rinsing with TBS, sections were immersed in Alexa 488 and 594 conjugated donkey IgG secondary antibody (1 : 500, Invitrogen) solution for 3 hrs at room temperature. After washing, sections were mounted on slides, air-dried, and coverslipped with mounting medium containing DAPI (Southern). Finally, slides were examined immediately and preserved in -20°C freezer for further image acquisition.

Brain sections were examined on a Leica DM5500B microscope. To quantify A11 DA-ergic neurons, TH^+^ neurons in a series of sections (210 *μ*m apart) were counted. TH^+^ neurons distributed in the posterior hypothalamus, dorsal to the mammillary bodies, immediately lateral to the third ventricle, and medial to the mammilotegmental tract (Bregma: -1.9 to -3 mm) were defined as A11 DA-ergic neurons. Quantification of A11 neurons was performed throughout the rostrocaudal extent of this region under a 40x objective. In all cases, quantification of cells was performed by a blind observer. Representative figures showing A11 neurons were obtained by a digital camera (Hamamatsu) and SlideBook software (Intelligent).

### 2.7. Statistics

Statistical analysis was performed using Prism 7.0 (GraphPad). An unpaired Student's *t*-test was used to compare the genetic alterations between 2 groups. The significance of variations between multiple experimental variables was calculated by a one-way ANOVA followed by Dunnett's multiple comparisons test. A difference was considered significant as *p* < 0.05. Data are represented as mean ± SEM.

## 3. Results

### 3.1. mRNA Levels of TH, DDC, and D_2_ Receptors Are Transiently Reduced in the Rostral Spinal Cord

**Figure 2:**
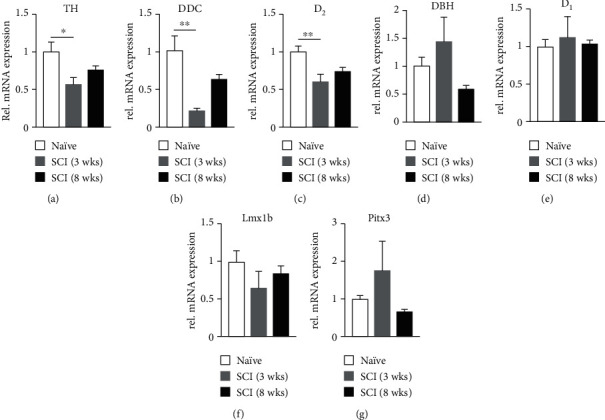
Alterations of DA-related mRNA levels in the spinal cord rostral to the injury site. Normalized qPCR results illustrate the change of gene expression in the T5 spinal segment. There is a significant reduction in mRNA levels of TH (a), DDC (b), and D_2_ (c) 3 weeks after SCI, but the level of these genes recovers at 8 weeks. In contrast, no change is observed in DBH, D_1_, Lmx1b, and Pitx3 at either time point (d–g). The results suggest that axonal DA synthesis is transiently impaired in the spinal cord rostral to the lesion site. ^∗^*p* < 0.05, ^∗∗^*p* < 0.01.

Three weeks after injury, qPCR analysis detected a significant decrease in mRNA levels of TH, DDC, and D_2_ in the T5 spinal cord segment above the lesion in comparison to naïve animals. However, at 8 weeks postinjury, there were no significant changes in expression of these genes (Figures 2(a)–2(c); *n* = 7 per group, one-way ANOVA followed by Dunnett's multiple comparisons test; TH: naïve vs. SCI 3wks, *p* = 0.012, 8 wks, *p* > 0.05; DDC: naïve vs. SCI 3 wks, *p* = 0.0012, 8 wks, *p* > 0.05; D_2_: naïve vs. SCI 3 wks, *p* = 0.0054, 8 wks, *p* > 0.05). The data indicate that DA-related gene expression in the spinal cord rostral to the lesion site is transiently reduced but subsequently recovered at the chronic stage after SCI.

Since TH and DDC are also related to norepinephrine (NE) expression, mRNA levels of DBH, which catalyzes DA to NE, were evaluated. The results did not show a significant change after SCI (Figure 2(d); *p* > 0.05, *n* = 7 per group, one-way ANOVA), suggesting that altered TH and DDC mRNA expression is largely related to alterations in the DA-ergic system. In all groups, mRNA levels of D_1_ receptors remained stable (Figure 2(e); *p* > 0.05). As D_1_ receptors are located postsynaptically [24], this indicates that postsynaptic neurons do not modify their expression of D_1_ receptors in response to altered DA signaling following SCI. The two transcription factors, Lmx1b and Pitx3, regulate the expression of TH and DDC in the cell nucleus rather than axons [25]. SCI did not induce changes in expression of these two factors in the upper thoracic spinal cord segment (Figures 2(f) and 2(g); *p* > 0.05). Together, the results indicate that the capacity for remaining A11 axons to produce DA rostral to the lesion site is transiently hindered after axotomy.

### 3.2. mRNA Levels of DA Synthesis Enzymes and D_2_ Receptors Decrease in the Caudal Spinal Cord

**Figure 3:**
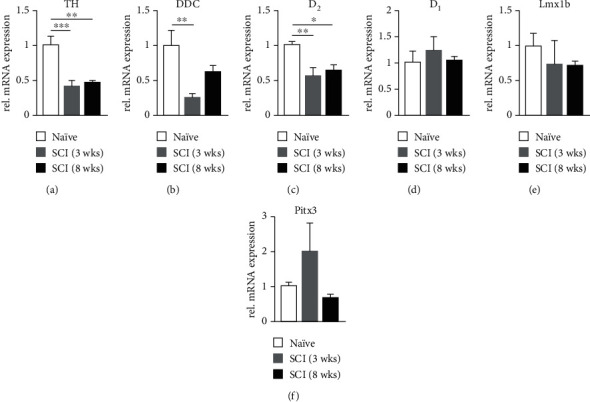
Changes of DA-related transcripts in the spinal cord caudal to the injury site. In the L1 spinal segment, mRNA levels of TH (a) and D_2_ (c) are downregulated 3 weeks after SCI, and this change is sustained out to 8 weeks. However, mRNA levels of DDC (b) are decreased 3 weeks after SCI but steadily recover at the 8-week timepoint. No changes were observed in D_1_, Lmx1b, and Pitx3 gene expression levels (d–f). ^∗^*p* < 0.05, ^∗∗^*p* < 0.01, ^∗∗∗^*p* < 0.001.

Examination of mRNA levels in the L1 spinal segment caudal to the injury showed that both TH (Figure 3(a); *n* = 7 per group, one-way ANOVA followed by Dunnett's multiple comparisons test; naïve vs. SCI 3 wks, *p* = 0.0004, 8 wks, *p* = 0.0012) and D_2_ receptor (Figure 3(c); naïve vs. SCI 3 wks, *p* = 0.0032, 8 wks, *p* = 0.0145) gene expression was significantly decreased at both 3 and 8 weeks after SCI, consistent with previous observations of degenerated TH^+^ projections below the lesion site [21]. Interestingly, the levels of DDC mRNA (Figure 3(b); naïve vs. SCI 3 wks, *p* = 0.0032, 8 wks, *p* > 0.05) were initially decreased at 3 weeks but returned baseline levels at 8 weeks following SCI. In addition, mRNA levels of D_1_, Lmx1b, and Pitx3 were not altered (Figures 3(d)–3(f); all *p* > 0.05).

### 3.3. mRNA Levels of DA-Related Genes and the Number of Neurons in the A11 Nuclei Remain Unchanged

**Figure 4:**
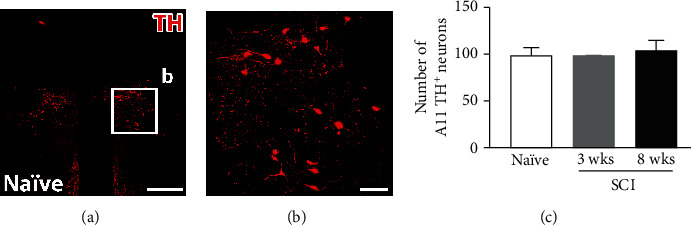
Quantitative analysis of A11 neurons between naïve and SCI rats. (a, b) A representative image illustrating the distribution of TH^+^ (red) neurons and their projections in the A11 region of naïve rats. (c) Quantitative analysis shows no significant difference in the number of TH^+^ neurons after SCI-induced axotomy vs. intact rats. (b) is boxed regions in (a). Scale bars: (a) 500 *μ*m; (b) 100 *μ*m.

Immunohistochemistry for TH was conducted to determine the possible change in the amount of A11 DA-ergic neurons. However, the number of TH^+^ cells in this region had no significant difference between the naïve and SCI rats at both 3 and 8 weeks (Figure 4; naïve 98 ± 9.02, 3 weeks 98 ± 1.16, 8 weeks 103 ± 12.50, all *p* > 0.05, one-way ANOVA; *n* = 3 per group). This indicates that SCI does not induce apoptosis of A11 DA-ergic neurons.

**Figure 5:**
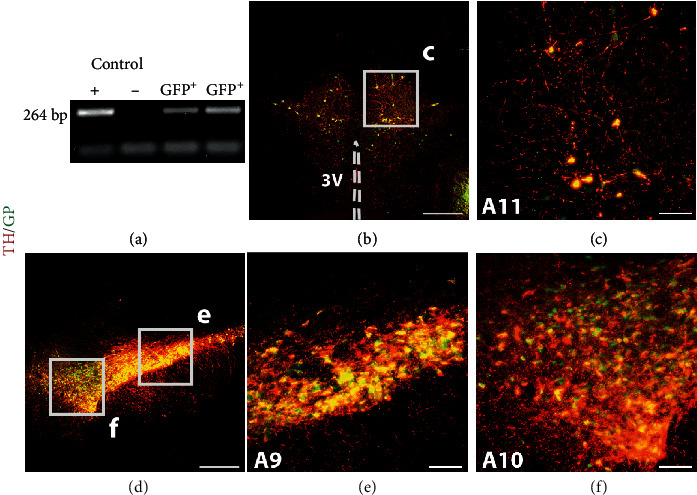
Characterization of an hTH-eGFP transgenic rat line. (a) An electrophoresis image shows the genotyping results of hTH-eGFP pups. From left to right, loading samples are amplified PCR products of the GFP^+^ breeding colony (positive control, +; lane 1), wild type rats (negative control, -; lane 2), and testing samples (lanes 3-4). (b–f) Colocalization of TH and GFP in A9, A10, and A11 nuclei. Immunostaining shows GFP-tagged TH^+^ neurons in the A11 (b, c), A9 and A10 nuclei (d–f). Scale bars: (b, d) 500 *μ*m; (c, e, and f) 100 *μ*m; V: ventricle.

Before single-cell LMD, colocalization of TH and GFP was validated by immunohistochemistry in the A9, A10, and A11 areas (Figure 5). Approximately 90% of TH^+^ neurons in the A11 region expressed GFP, while 15% of GFP-expressing neurons did not colabel with TH^+^ [26]. As for gene expression in the neuroplasm, Pitx3 and Lmx1b mRNA levels were undetectable in LMD-isolated A11 DA-ergic neurons. Alternatively, another DA-related transcription factor Nurr1 was assessed. Results revealed that there was no significant alteration 3 weeks after SCI in any of the genes tested which included TH, DDC, D_2_, VMAT2, and Nurr1 (all *p* > 0.05, unpaired *t*-test; *n* = 6 in naïve, *n* = 5 in SCI). The data indicates that SCI does not affect the expression of DA-related gene transcripts in A11 neuronal soma.

### 3.4. Alterations of DA-Related Genes in the A9 and A10 Nuclei after SCI

**Figure 6:**
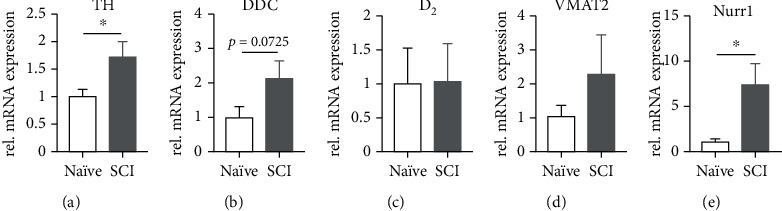
Transcripts of classical DA-ergic enzymes increase in A9 and A10 neurons after SCI. mRNA levels of TH (a) and the transcription factor Nurr1 (e) are upregulated in the A9 and A10 nuclei 3 weeks after SCI. The DDC transcript shows a trend towards significance (b), while D_2_ (c) and VMAT2 (d) do not change. ^∗^*p* < 0.05.

DA neurons within the A9 and A10 nuclei project to structures within the brain rather than the spinal cord [6]. To exam possible indirect influence of SCI on these regions, we assessed gene expression of DA neurons in A9 and A10 nuclei. Interestingly, mRNA levels of TH (Figure 6(a); naïve vs. SCI, *p* = 0.036; *n* = 6 in naïve, *n* = 5 in SCI; unpaired *t*-test) and the transcription factor Nurr1 (Figure 6(e); naïve vs. SCI, *p* = 0.021) increased 3 weeks after SCI. Additionally, DDC mRNA levels, although not significant, trended toward an increase in expression (Figure 6(b); naïve vs. SCI, *p* = 0.072). However, there was no change in expression of D_2_ and VMAT2 (Figures 6(c) and 6(d); all *p* > 0.05). The results suggest that DA neurons in A9 and A10 nuclei increase gene expression of enzymes for DA synthesis but may not change the ability of DA packaging and autoregulation after SCI.

## 4. Discussion

**Figure 7:**
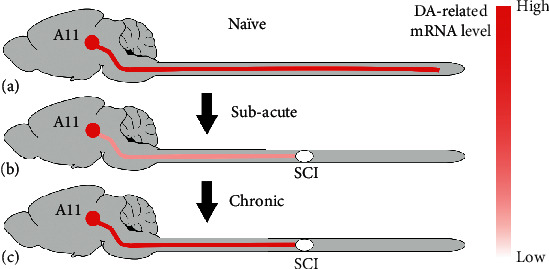
A diagram illustrating the dynamic changes of DA-related mRNA levels in A11 diencephalospinal pathways after SCI. Diencephalic A11 neurons project to all levels of the spinal cord. The majority of DA-related transcripts are transiently reduced in the spinal cord rostral to the injury site, while they are consistently decreased in the caudal spinal cord. In the chronic phase, remaining A11 diencephalospinal pathways undergo restoration of DA-related transcripts in the rostral spinal cord. Notably, the number of A11 DA-ergic neurons and DA-related mRNA levels in the neuroplasm do not change after SCI.

A11 neurons are the sole source of supraspinal transportation of DA to the spinal cord. In the present study, we found transient changes of DA-related genes in the spinal cord rostral to the lesion and long-term changes caudal to the lesion after SCI. In contrast, neither the number of A11 DA-ergic neurons nor DA-related mRNA gene expression levels in the neuroplasm was affected (Figure 7). Thus, injury transiently alters mRNA levels of DA-related genes in the remaining A11 axons above the lesion site. These dynamic changes can further be associated with the axtomoty of descending DA-ergic pathways.

We reported a significant decrease in mRNA levels of TH, DDC, and D_2_ in the spinal cord rostral to the lesion 3 weeks after SCI and recovery at 8 weeks. Specific genes were chosen to assess dynamic changes in A11 DA-ergic neurons and spinal cord tissue for several different reasons. Firstly, Lmx1b, Pitx3, and Nurr1 are transcription factors that regulate TH and DDC expression. However, it is important to note that these 3 factors do not exclusively exist in DA-ergic neurons [25]. In addition, Nurr1 was also examined given that it regulates the expression of both DA synthesis enzymes (TH and DDC) and also microglia-involved inflammatory mediators [27, 28]. Considering that SCI induces massive neuroinflammation, we examined mRNA levels of Lmx1b and Pitx3 but not Nurr1 in the rostral spinal cord while all three were measured in dissected A11 neurons. As anticipated, no detectable changes in these factors were revealed in the spinal cord. Further, the postsynaptic DA-ergic receptor D_1_, and post- and presynaptic D_2_ autoreceptor were included in analyses due to their involvement in the regulation of DA synthesis, release, and reuptake [24]. Therefore, a transient decrease of these transcripts may suggest that axonal DA synthesis is temporarily compromised in descending A11 axons following SCI. Meanwhile, DBH mRNA levels remained unchanged. It is possible that noradrenergic projections from the locus coeruleus and A5 region to the spinal cord underwent fast recovery following SCI, and alterations in expression were not detected accordingly [29]. Because noradrenergic projections also contain TH and DDC mRNA, the transient decrease of DA-related transcripts observed in the rostral spinal cord could represent the conditions in A11 fibers if their levels sustain in noradrenergic projections as DBH.

To determine if transiently suppressed expression of DA-related mRNA in the rostral spinal cord is due to changes in the soma, we quantified the neuronal number and analyzed gene expression levels in the cytoplasm of A11 cells. Consequently, no changes were detected in either of these experiments which suggests that short-term suppression is restricted in axons rather than neuronal bodies. In fact, there are several possibilities that could explain the transient changes in expression of TH, DDC, and D_2_. For instance, altered axonal mRNA transportation rates after SCI could support the temporal changes seen in gene expression levels in A11 axons above the lesion site. Previous studies reported a lack of fast axonal transport after injury, and this deficiency contributes to the failure of central axonal regeneration [30]. Another possibility is the remodeling of axonal mRNA composition after axotomy. Specifically, it has been reported that mitochondrial mRNA is downregulated in injured axons but upregulated in those associated with axonal targeting and synaptic formation [31]. Thus, it is plausible that DA-related transcripts are transiently reduced to accommodate the priority of axonal and synaptic plasticity. Once neuronal homeostasis is achieved in the chronic phase, these neurons regain the ability to synthesize DA in the rostral spinal cord. Lastly, decreased axonal mRNA may reflect a reduced density of projection branches. Following SCI, spontaneous axonal sprouting and pruning occurs rostral to the lesion site [16, 32, 33]. Injury-induced plasticity often facilitates new synapse formation onto long-range propriospinal interneurons.

Traumatic SCI causes axonal degeneration below the lesion [34]. After a complete T10 spinal cord transection, A11 fibers gradually degenerate, and mRNA levels of TH and D_2_ decrease in the L1 spinal segment at both the subacute and chronic stages. However, a transiently reduced level of DDC was detected. The recovery of DDC transcript in the chronic phase is in line with other reports that the ability of DDC to synthesize DA is enhanced in pericytes of blood vessels and spinal DDC^+^ neurons following SCI [35–37]. Similar to previous findings in mice with a lesioned A11 region, we revealed decreased D_2_ receptor expression, while D_1_ mRNA levels remained unchanged in the caudal spinal cord. Since D_2_ is also an autoreceptor that is expressed on presynaptic DA-ergic neurons, alterations of D_2_ expression are likely due to A11 axonal degeneration [8]. Although postsynaptically located D_1_ receptor expression did not change as indicated by assessment of whole tissue of the caudal spinal cord, one cannot exclude the possibility of upregulation of this receptor in some specific neurons related to autonomic or somatic function.

One unique characteristic of axonal protein synthesis is that axonal mRNA allows precise spatiotemporal regulation of axonal proteome in response to input from extracellular environment. Accordingly, reduced axonal DA-related transcripts hinder DA release when sudden stimuli require a large amount of DA in a short period of time. Certainly, further experiments would need to be conducted to provide direct evidence of DA release to confirm this assumption. It was shown that DA inhibits pain perception through D_2_ receptor-mediated pathways [38]. SCI often induces neuropathic pain above the lesion that is characterized by mechanical allodynia and heat hyperalgesia in the forelimbs [39, 40], which could be associated with compromised A11 DA-ergic pathways [38]. The transient decrease of DA-ergic enzyme mRNA levels that were observed in the present study may underlie the pathology of neuropathic pain in the subacute phase of SCI. If this is the case, DA-ergic machinery could become a potential therapeutic target for SCI-induced pain. Additionally, visceral pain may be related to SCI-induced autonomic dysfunction, such as disordered bladder and bowel activity and autonomic dysreflexia [41, 42]. Therefore, pharmacological modulation of DA signaling could be an appropriate treatment approach to augment various pelvic visceral complications following SCI.

Though A9 and A10 neurons extend their projections within the brain, previous studies showed that SCI or peripheral nerve injury can change the firing properties of these neurons as well as the release of DA. This suggests an indirect effect of injury on these neurons, such as adaptation or neuroinflammation [43–45]. Following SCI, the increase of gamma-aminobutyric acidergic (GABAergic) neuronal activity in the A10 region suppresses DA neuronal activity and further decreases DA release [43]. A proinflammatory cytokine, macrophage migration inhibitory factor (MIF), downregulates DA release after peripheral nerve injury [45]. Collectively, upregulated DA-related genes may be a compensatory response to the deficient release of DA in A9 and A10 nuclei. On the other hand, dysregulation of A10 neurons is often implicated in addiction, depression, and stress [46]. Patients with SCI are twice as likely to also suffer from depression [47]. Whether this is related to changes seen in the A10 region remains unclear and will require further studies to address this question.

Due to technical limitations, mRNA levels could easily be detected in the cell body and cytoplasm as opposed to axons. While *in situ* hybridization is a common method used to visualize the location of mRNA, it is difficult to detect transcripts in axons due to low sensitivity [48]. Microarrays and real-time qPCR are, however, sensitive enough to detect low levels but it is hard to isolate axons without contamination from surrounding nonneuronal tissue [49]. This causes inevitable nonaxonal components to also be included in qPCR data. Based on previous reports, DDC is expressed in blood vessel pericytes, spinal DDC^+^ neurons, and astrocytes [35, 50]. Accordingly, all these factors could contribute to the recovery of DDC expression in the caudal spinal cord at the chronic stage. SCI causes microglia activation, gliogenesis, and macrophage infiltration in the spinal cord [51, 52]. Because subtle levels of TH, DDC, and D_1/2_ receptors can be detected in macrophages or microglia [53, 54], the observed changes could be influenced by both macrophage and microglia contamination within our samples. Despite the fact that we cannot fully exclude the involvement of these nonneuronal cells, their contribution is trivial to our results. SCI-induced inflammation occurs in the rostral and caudal spinal cord. Thus, it is expected that reactive immune cells would have the same genetic alterations throughout the cord. Yet, different changes in DA-related gene expression were detected between the rostral and caudal spinal cord segments of the lesion site, suggesting this difference mainly originated from specific components instead of general immunoreaction. Rationally, alterations in DA-related genes rostral to the lesion site are attributable to dynamic changes of A11 axons and those caudal to the lesion are due to degeneration of these projections. It is important to note that some results have a trend toward statistical significance but the *p* value is not in fact significant, which may have resulted from a type II error.

## 5. Conclusions

In this study, midthoracic SCI causes a temporal decrease of DA-related mRNA levels in the spinal cord rostral to the lesion site, whereas A11 DA-ergic neurons do not undergo apoptosis nor do they alter gene expression levels in the soma. Overall, the data suggests that remaining A11 diencephalospinal pathways transiently reduce the ability to synthesize DA following SCI.

## Figures and Tables

**Table 1:** Primers used in this study.

Primer name	Forward primer sequence (5′ ➔ 3′)	Reverse primer sequence (5′ ➔ 3′)
eGFP ORF	CAGCACGACTTCTTCAAGTCC	GATCTTGAAGTTCACCTTGATGC
MAP2	GCCAGCCTCAGAACAAACAG	AAGGTCTTGGGAGGGAAGAAC
GFAP	CCCCATTCCCTTTCTTATGC	ATACGAAGGCACTCCACACC
CD11b	GGCTCCACTTTGGTCTCTGT	GACCACCTCCTGCTTGTGAG
CNPase	AAATGGCAGACCAGTATCAGTACC	GTCTCAGAACTCTTTTTGGTCAGG
18s	AAGTCCCCTAACACCCTCGT	AGTTTGTGGAAGAGCGGAAG
TH	CCACGGTGTACTGGTTCACT	GGCATAGTTCCTGAGCTTGT
DDC	GAAGATGCTTGAGCTGCCAG	GAATCTGCAAACTCCACGCC
DBH	GAGGCGGCTTCCATGTACG	TCCAGGGGGATGTGGTAGG
Lmx1b	TTCCTGATGCGAGTCAACGAG	TCCGATCCCGGAAGTAGCAG
Pitx3	CCTACGAGGAGGTGTACCCG	ACCGAGTTGAAGGCGAACG
Nurr1	GGTTTCTTTAAGCGCACGGTG	TTCTTTAACCATCCCAACAGCCAG
D_1_	AACTGTATGGTGCCCTTCTGTGG	CATTCGTAGTTGTTGTTGCCCCG
D_2_	ACCTGTCCTGGTACGATGATG	GCATGGCATAGTAGTTGTAGTGG

## Data Availability

All data used to support the findings of this study are included within the article.
